# Preparation of ultra-high mechanical strength wear-resistant carbon fiber textiles with a PVA/PEG coating

**DOI:** 10.1039/d1ra03983k

**Published:** 2021-07-23

**Authors:** Ziqin Feng, Feng Hu, Leifeng Lv, Li Gao, Hailin Lu

**Affiliations:** Group of Mechanical and Biomedical Engineering, Xi'an Key Laboratory of Modern Intelligent Textile Equipment, College of Mechanical & Electronic Engineering, Xi'an Polytechnic University Xi'an Shaanxi 710048 P. R. China lu@xpu.edu.cn; Department of Orthopadics, The Second Affiliated Hospital of Xi'an Jiaotong University, Xi'an Jiaotong University Xi'an Shaanxi 710061 P. R. China 15529351957@163.com; Department of Gynaecology and Obstetrics, The First Affiliated Hospital of Xi'an Jiaotong University, Xi'an Jiaotong University Xi'an Shaanxi 710061 P. R. China gaoli23@mail.xjtu.edu.cn

## Abstract

Polyvinyl alcohol (PVA) is an organic polymer that is non-toxic, harmless to the human body, and has good biocompatibility. Polyethylene glycol (PEG) is a polymer that has good lubricity and compatibility. The unique graphite structure of carbon fibers can promote the potential application of carbon–fiber composites in tribology. This study explores the relationship between two kinds of organic polymer compounds and carbon fiber cloth (CFC), specifically a PVA/PEG composite coating that is impregnated on the CFC surface. The CFC is synthesized by chemical cross-linking, and the CFC composites (PVA/PEG/CFC) were synthesized. The tribological properties of PVA/PEG/CFC were tested under different concentrations, loads, and velocities. The effects of the different lubricants, surface morphologies, and tensile strengths on the mechanical and tribological properties of PVA/PEG/CFC were studied. In comparison to the original CFC, the friction coefficient and wear morphology of the composite material were reduced and the friction coefficient trend was stable. The addition of PVA/PEG improved the surface lubrication performance of the composite material and reduced the average friction coefficient. In addition, under the different lubrication mechanisms, oil as a lubricant can significantly reduce the friction coefficient and surface wear. In summary, the biocompatible coating process that is proposed in this study can effectively improve the tribological properties of the surface of the CFC.

## Abstract 2

Polyvinyl alcohol (PVA) is an organic polymer that is non-toxic, harmless to the human body, and has good biocompatibility. Polyethylene glycol (PEG) is a polymer that has good lubricity and compatibility. As a new coating material, PVA/PEG has good mechanical properties.

## Graphical abstract

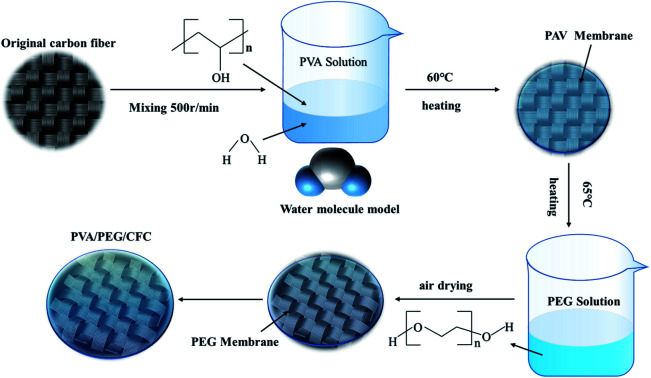

## 1 Introduction

Carbon Fiber Cloth (CFC) is a unidirectional carbon fiber reinforcing product that is usually made of 12k carbon fiber silk. In the past few decades, with the continuous development of the CFC industry, an increasing number of industries and enterprises have applied CFC. CFC is mainly used for tensile, shear, and seismic reinforcement of structural components, and it is used with a high polymer to become a carbon fiber composite material.^1,2^ Studies have shown that carbon fiber composite materials are lightweight, they have a high specific strength, modulus, and durability, and they have wide application prospects in sporting goods, medical instruments, aerospace, and other industrial fields.^3–10^ However, by increasing the number of carbon fiber composites that are used, a variety of new polymer materials can be developed, which leads to the accumulation of some non-degradable polymers, such as waste accumulation during manufacturing and maintenance.^11,12^ Rapid development manufacturing is currently a serious environmental problem. Presently, manufacturing development emphasizes green sustainable development; hence, these environmental problems can be solved. Most polymers are used as thermosetting materials and they have an inherent three-dimensional crosslinked network structure, which has a high thermal stability and chemical resistance,^13^ thus, polymers are difficult to degrade and melt. Therefore, by considering the environmental protection problem, selecting a polymeric material is a characteristic of this study.

From one investigation, it was determined that the surface inertia of the carbon fiber is large, and the physical and chemical forces between the carbon fiber and the polymer matrix are weak. The carbon fiber consists of graphitized carbon because of its unique crystal structure; therefore, the surface lubrication performance is good.^14,15^ However, the untreated carbon fiber surface produces small and weak graphite crystals during graphitization, which results in a small surface activity ratio and leads to a lower surface energy and surface hydrophobicity.^16,17^ As a result, the surface of the untreated carbon fiber is difficult to wet, and the polymer and carbon fiber surfaces do not bond adequately.^18,19^ It was discovered that the surface of the carbon fiber can be modified by performing chemical oxidation^20^ with a plasma^21^ and applying a coating.^22^ This can achieve better interface bonding and mechanical properties of the carbon fiber.^23^ Some researchers have studied the impregnation solution on the surface of the fabric to enhance its mechanical properties. For example, Tan *et al.*,^24^ prepared a silica sol suspension coating on the surface of a CFC to enhance the strength of the fabric, and the experimental effect was remarkable. He and Chen *et al.*,^2^ studied the application of a flexible carbon cloth coating that was derived from biomass to prepare a supercapacitor material, which showed good stability. Galyshev *et al.* proposed a combination method of sol–gel technology and electrochemical deposition to prepare a silica coating on carbon fibers. The effects of current density, deposition time, and salt concentration on the structure and thickness of the coating were studied.^25^ Zn *et al.* developed an ultrasonically fabricated silver/iota-carrageenan/cotton bio-nanocomposite as an efficient material for biomedical applications. They used an iota-carrageenan as a carbohydrate polymer to assemble AGNPs on cotton under the action of an ultrasonic wave and showed that this nanocomposite material has good thermal stability. It also has significant advantages for application in antimicrobial therapy and drug delivery.^26^

In recent years, numerous scholars have studied the surface modification of fabric and proposed a method of surface modification using polymer coating; for example, Alimohammadi *et al.* proposed and prepared polyvinylpyrrolidone/carbon nanotube/cotton nanocomposite (polyvinylpyrrolidone/carbon nanotube/cotton) coated cotton fabric and studied the functionality and durability of the composite.^27^ Gashti *et al.* prepared a polyvinyl phosphonic acid/carbon nanotube composite coating to induce flame-retardant cellulose fibers with ultraviolet radiation. Carbon nanotubes were deposited on the surface of cotton using vinyl phosphonic acid monomer as the crosslinking agent and benzophenone as the catalyst. The effects of polymer materials on the thermal and combustion properties of cellulose fibers were studied.^28^ Although many polymers have been used as coatings to enhance the mechanical properties of fabric composites, the selection of polymer materials remains a challenge because it is difficult to break down many polymer materials, resulting in environmental pollution. However, considering the relevant information, when the current use of a biocompatible solution coating on the CFC surface is taken into account, there are relatively few studies in the literature. PVA is a biodegradable polymer that is harmless to the human body and it has good biocompatibility, especially in the medical field, since it has a wide range of applications.^29,30^ It is a green and environmentally-friendly material, which is often used as a fabric treatment agent. It can also be used to coat the CFC surface to enhance the mechanical properties of the surface. PEG is a high polymer that is non-irritating and it has good water solubility. It is an excellent lubricant and the moisture material can be used as a crosslinking agent, it can improve the surface lubricity of the CFC, and the polymer easily degrades. As a result, this can be used to solve environmental pollution problems. Therefore, this study mainly uses non-toxic and harmless polymer material as the surface coating of CFC.

In this study, the most significant aspect is the use of good film forming characteristics of PVA. PEG is used as a lubrication filling material; PVA/PEG was applied to the surface of CFC by chemical crosslinking method. A layer of lubricating film was formed on the surface of CFC to improve the friction and wear performance of CFC surface. Finally, the synthetic PVA/PEG/CFC composites were prepared, and then the mechanical properties of the composites were verified through a series of friction tests and tensile tests. The PVA/PEG/CFC composites were analyzed by performing the following techniques: Fourier transform (FTIR), X-ray diffraction (XRD), thermogravimetric (TGA), and X-ray photoelectron spectroscopy (XPS). The experimental results showed that the addition of PVA/PEG improves the surface lubrication performance of the composites and the surface wear is reduced, in which the PVA/PEG/CFC composites display better mechanical properties. The preparation process of the solution is simple, which greatly reduces the preparation cost and improves the production efficiency compared with the cumbersome coating process used earlier and has good application prospects.

## 2 Experimental

### 2.1 Materials

PVA (polymerization degree, 1750; hydrolysis degree, 99%) and PEG (molecular content: 4000) were purchased from the Tianjin Demo Chemical Reagent Factory. CFC was supplied from Xi'an Aerospace Composite Research Institute, and the specific mechanical indexes are shown in Table 1. In this study, all the materials and reagents consisted of an analytical grade and they were used as received; they were not further purified. Where, k represents the original number, and 1k represents one strand of filaments which contains 1000 filaments.

**Table 1:** Mechanical index of the CFC fiber raw material

Type	Preparation method	Grammage (g m^−2^)	Tensile strength (GPa)	Breadth (cm)	Required thickness (mm)
3k	Plain woven	240	3.5	100	0.2713

### 2.2 Preparation of the samples

**Fig. 1:**
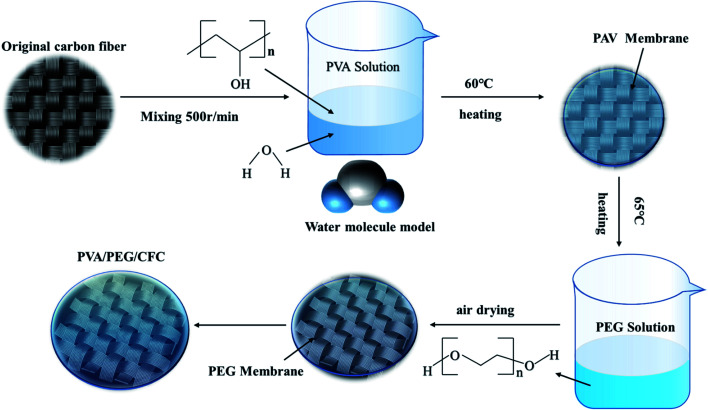
Preparation process of PVA/PEG/CFC.

First, the CFC was cut into 50 cm × 50 cm cubes as a backup material. 10 g of PVA was added to 90 ml of distilled water and stirred at 200 °C and 500 rpm for 30 min to prepare a 10% PVA solution,^31^ according to the relevant data; PVA has better mechanical properties at this concentration. It was then soaked in the 65 °C solution and then removed to dry. PEG solutions with different concentrations were prepared, and different weights of PEG were added to distilled water in different volumes to obtain different concentrations of the PEG solutions (10, 20, 30, 40, 50, and 60 wt%). The dried carbon cloths were immersed in different concentrations of PEG solutions and then placed in an incubator at 60 °C, after which they were removed and dried. Finally, PVA/PEG/CFC composites were prepared by this method of repeatedly invading the coating. The preparation process is shown in Fig. 1. The coating thickness of the prepared PVA/PEG/CFC composite was measured using a micrometer (SH1906A1999). Measurements were performed in the same plane at different points ten times, and then, the average value was taken. The final coating thickness was 0.0628 mm. Unless otherwise stated, the following references to PVA/PEG/CFC refer to 10% of PVA and 30% of PEG.

### 2.3 Characterization

The friction properties were tested with a GSR-2 friction testing machine (China rubber alcohol friction testing machine). A reciprocating friction driving system was used to slide on the PVA/PEG/CFC surface and the slider balls were steel balls (Φ9.95). Before each test, the steel ball was cleaned with acetone, and the steel ball was mounted on the friction rod for the friction test. During the testing process, the friction and wear tests were carried out under different lubrication environments, loads (5–10 N), and velocities (0.5–2 cm s^−1^) to better observe the experimental results. Each test was repeated three times with an average friction time of 20 min per time. Finally, the average value was taken as the friction result analysis, and the wear of the composites was observed by electron microscopy. In addition, the fixed load was 5 N and the speed was 1.0 cm s^−1^ in the following text, unless otherwise specified. All the material specifications were provided by the manufacturer. Three materials were placed in an appropriate amount of PBS solution, and a gel solution with a mass volume fraction of 100 mg ml^−1^ was formed after a high temperature and high pressure. The complete cell medium components were as follows: 10% fetal bovine serum (Gibco), 100 U ml^−1^ penicillin–100 μg ml^−1^ streptomycin (Gibco), and RPMI1640 (Gibco). After that, the resuscitation of the third generation of L929 cells was used for the experiment. When the cells grew to about 85%, the medium was discarded, the sterile PBS solution was rinsed twice, and trypsin (GBICO) was added for digestion for about 1 min. After the digestion stopped in the complete medium, the solution was resuspended and centrifuged, the supernatant was discarded, and the complete medium was added to the cell precipitation to make the cell suspension. The cell suspensions were mixed and counted, and the cell density was 10 000 cells per well and inoculated in 96-well plates with a total volume of 150 μl in each well. 10 μl of the corresponding material was added to each well, and then they were divided into four groups according to the added material: PEG, PVA, PVA–PEG, and the blank control groups, with six wells in each group. The culture was continued at 5% CO_2_ and 37 °C for 24 h, after which the material and medium were discarded, and gently washed in sterile PBS solution two times. The complete medium that contained 10 μl of CCK8 solution was added to each well, and the culture continued for 1 h. The solution was fully mixed by shaking at a low speed for 10 min. The OD value of each well was detected by using FLUO Star Omega at 450 nm, and the data were recorded and analyzed. The surface chemical changes of the samples and their specific interactions were studied by performing Fourier transform infrared spectroscopy (FT-IR) and the samples were scanned with a Nicolet IS50 spectrometer (Thermo Fisher Scientific, USA) at wavelengths that ranged from 500 cm^−1^ to 4000 cm^−1^. The crystal phase structure was characterized by XRD in the scanning range of 5–75° with a Bruker D8 advanced diffractometer (Bruker, Germany). The constituent elements and chemical binding forms of the materials were identified by X-ray photoelectron spectroscopy (XPS, Escalab250XI, Thermo Fisher Scientific, USA). The tensile properties of the PVA/PEG/CFC composites were tested with a tensile testing machine (ZQ-218), cut into 50 cm slices with a 10 cm width, and the contact parts between the two ends of the samples and the fixture were wrapped with a thick tin foil to increase the friction. The specimens were subjected to tensile loading at a speed of 3 mm min^−1^ until they were broken and the failure load was recorded. A thermogravimetric (TGA) analysis and melting crystallization curve (DSC) were performed with a 409C thermobalance (NETZSCH Group, Germany) and tested in air atmosphere with a temperature range between 25 °C to 1000 °C.

## 3 Results and discussion

### 3.1 XPS and XRD analysis

**Fig. 2:**
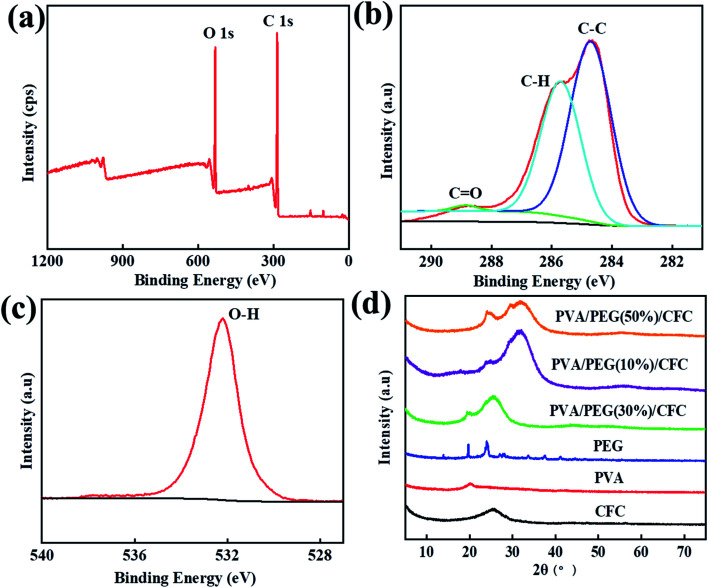
XPS analysis of PVA/PEG/CFC. (a)Wide-scanning spectra, (b) C 1s, (c) O 1s, and (d) XRD patterns of PEG, PVA, CFC, PVA/PEG(10%)/CFC, PVA/PEG(30%)/CFC, PVA/PEG(50%)/CFC.

XPS detection can be used to analyze the surface chemical properties of composite materials and measure the elemental composition of the materials, and surface elements and their corresponding functional groups can be qualitatively and quantitatively analyzed by the characteristic peaks in the spectra. XRD detection can be used to analyze the crystal structure of the composite material, through the analysis of the diffraction peak, we can know whether the PVA/PEG coating was effective on the surface of the CFC. Fig. 2(a) shows the surface chemical element composition of PVA/PEG/CFC that was measured by XPS, in which the peak values of the carbon and oxygen elements appeared between 281–291 eV and 526–540 eV. Fig. 2(b) shows the spectral analysis of C 1s, and the three peaks correspond to the C atoms of the different function groups C–C (284.7 eV), C–H (285.7 eV), and C=O (288.7 eV).^32^Fig. 2(c) is the spectral analysis diagram of O 1s, and a peak value corresponding to the O–H functional group appears at 532.2 eV. Finally, the XPS test showed that the surface of PVA/PEG/CFC composite material mainly contains two elements, C and O. Fig. 2(d) shows the XRD patterns of PVA, PEG, and PVA/PEG/CFC composites, and PVA/PEG/CFC has two diffraction peaks, which are 25.3° and 19.4°, respectively, which are similar to the characteristic peaks of PEG. However, the wide diffraction peak at 25.3° is caused by the superposition of CFC and PEG, so the diffraction peaks of PVA/PEG/CFC have a larger intensity and width, a characteristic peak of 19.8° is equivalent to PVA. A wide diffraction peak at 25.2° contributes to the CFC curve, which corresponds to the (002) plane of the CFC.^33^ From this, the interaction between PVA and PEG in the composites has an influence on the crystallinity of PVA/PEG/CFC.

### 3.2 TGA and FT-IR analysis and biological test

**Fig. 3:**
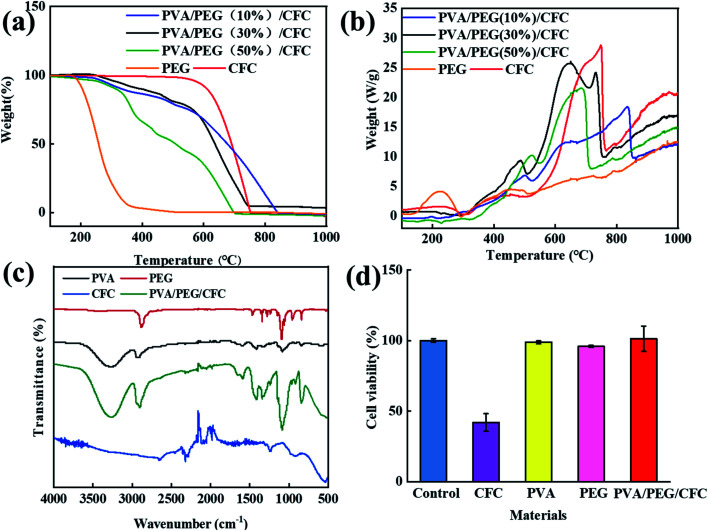
Analysis of PVA/PEG/CFC. (a) TGA, (b) DSC, (c) FTIR spectra of PEG, PVA, CFC, and PVA/PEG/CFC. (d) The cell viability of the different materials.

Fig. 3(a) The TGA analysis showed that the untreated carbon fiber fabric started to degrade at 600 °C, and the weight dropped sharply. The decomposition profile of the PVA/PEG/CFC composites began to degrade at 220 °C, and this continued to 720 °C. In comparison to the original carbon fiber fabrics, the composites have a lower thermal decomposition rate. This indicates that the presence of PVA/PEG improves the thermal stability of the composites.^31,34^ The thermal stability of PVA/PEG (30%)/CFC is between low concentration (10%) PEG and high concentration (50%) PEG. PEG is used as a control experiment, in order to show that the addition of PEG makes PVA/PEG (30%)/CFC composite material have better thermal stability. As shown in Fig. 3(b), the melting crystallization curve (DSC) of the PVA/PEG/CFC composites was determined by differential scanning calorimetry.^35^ For the PVA/PEG/CFC composites, it is shown that the decomposition ability is sharper and stronger at *T* = 500 °C and 620 °C. The original CFC, on the other hand, does not have a very sharp curve, the sharp peak value of the original CFC curve was reduced due to the addition of PEG. Therefore, in the temperature range of 30 °C to 650 °C, with an increase in the decomposition temperature, the cross-linking density increases and the interaction between the polymer chains increases, which indicates that the addition of PVA/PEG has a positive effect on the composites.^31^Fig. 3(c) shows the FT-IR spectra of PVA, PEG, CFC, and PVA/PEG/CFC. The absorption peak of PVA in the FT-IR diagram at 3000–3500 cm^−1^ is due to the stretching action of O–H, and the double peak at 2900 cm^−1^ is caused by the symmetric and antisymmetric stretching vibration of C–H in PVA. The peak at 1634 cm^−1^ is due to the presence of C=O in PVA,^36^ and the peak at 1158 cm^−1^ is mainly attributed to the relationship between the crystallinity of PVA and the carboxylic group stretching (C–O) vibration.^37^ The peak at 847 cm^−1^ is caused by the bending vibration at the C–H plane,^36^ the PEG FT-IR spectra at 1196 cm^−1^ shows a peak similar to the absorption peak of PVA, which indicates that the PEG peak may also be caused by the C–O–C asymmetric vibration. The absorption peak at 2950 cm^−1^ is caused by the C–H stretching vibration of CH_2_ in PEG,^37,38^ The peak values of PVA/PEG/CFC at 845.44 cm^−1^, 1087.45 cm^−1^, 2902.51 cm^−1^, and 3265.35 cm^−1^ are caused by the superposition of the peak values of CFC, PVA, and PEG. The peak value of CFC at 750 cm^−1^ is caused by the vibration of the C–H symmetric stretching. The peak value at 1200 cm^−1^ is caused by the stretching effect of C=O in CFC, and the characteristic peak value at 2210 cm^−1^ is caused by the stretching effect of C=C.^39^Fig. 3(d) shows the absorbance value (OD value) that was determined by an enzyme-linked immunoassay at 540 nm. In comparison to the control group, the cell growth of the untreated carbon fiber fabric was inhibited, and the cell growth of PVA, PEG, and PVA/PEG/CFC was barely affected. Therefore, the addition of PVA and PEG can improve the biocompatibility of the carbon fiber fabric since it greatly increases the survival rate of the cells.

### 3.3 The lubrication effect of the different PEG concentrations in PVA/PEG/CFC

**Fig. 4:**
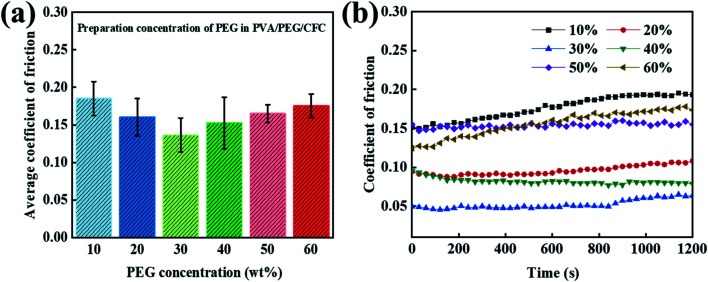
Curves of the prepared concentration of PEG in PVA/PEG/CFC. (a) The average coefficient of friction (b) and the coefficient of friction.

When exploring the friction properties, PEG has excellent lubricity and dispersion, and different concentrations of PEG also have different lubrication effects. To study the excellent lubrication effect, six groups of contrast curves with different concentrations were prepared. Fig. 4(a) shows a 20 minute period, where the average friction coefficient graph of the different concentrations of PEG was added to the PVA/PEG/CFC composites. It can be observed from the graph that the average friction coefficient first decreases and then it increases with an increasing PEG content. In addition, the average friction coefficient of the carbon fiber composites is the lowest when the content reaches 30% (10% PVA/30% PEG). Fig. 4(b) shows the corresponding figure's friction coefficient, and adding different PEG contents significantly lowers the friction coefficient of CFC. The addition of PEG can form a lubrication film on the CFC surface, which can greatly reduce the friction and wear. However, by increasing the friction time, when considering the slightly stable friction coefficient at a 30% concentration, the total friction coefficient is the lowest. When its value is 0.05, which is approximately one-third of the value at a low concentration (10%), the experimental surface lubrication effect is good.

### 3.4 The lubrication effect of the different loads in PVA/PEG/CFC

**Fig. 5:**
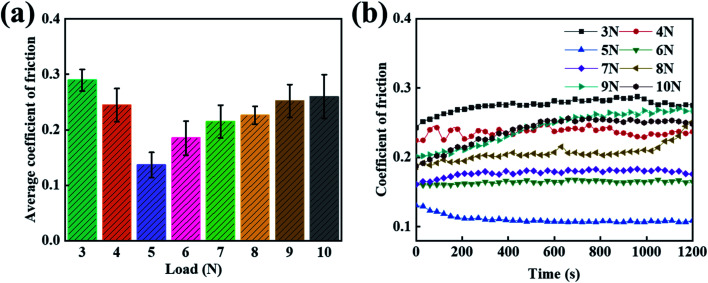
Curves of the different loads of PVA/PEG/CFC. (a) The average coefficient of friction. (b) The coefficient of friction.

It was previously reported that the coating effect at a 30% PEG concentration has a better lubrication performance than the other concentrations. However, since there are many applications of carbon fiber composites, the single load condition is insufficient to verify whether the friction and wear can be effectively improved. In order to study the friction properties of the composites under harsh conditions, under fixed conditions, the average friction coefficient of the PVA/PEG/CFC composites under different loads was tested. As shown in Fig. 5(a), the chart shows that as the load increases, the average friction coefficient of the PVA/PEG/CFC composite decreases and then increases. When 5 N is applied to minimize the average friction coefficient, the value of the average friction coefficient is approximately 0.12. This is due to the increase in the load under the condition of a constant pressure on the carbon fiber surface, in which the lubrication film can release PEG. Because of the PEG self-lubrication, the average friction coefficient of the composite decreased. With a further increase in the load, this results in the accumulation of friction heat on the composite material friction interface and CFC surface lubrication membrane softening. By increasing the temperature, which results in damage of the friction lubricating film, this causes friction and the CFC has direct contact with the sphere when friction is present. Therefore, the average friction coefficient of the PVA/PEG/CFC composites increases again. Fig. 5(b) shows the friction coefficients of the PVA/PEG/CFC composites. With the increase in the friction time, the friction coefficients of the different loads also relatively increase, but the overall curve is relatively stable. This is demonstrated in which the coefficient of friction of the composite material is the highest under the conditions of low and high loads. The data demonstrates that the PVA/PEG/CFC composite material is not suitable for use under harsh conditions.

### 3.5 The lubrication effect of the different speeds in PVA/PEG/CFC

**Fig. 6:**
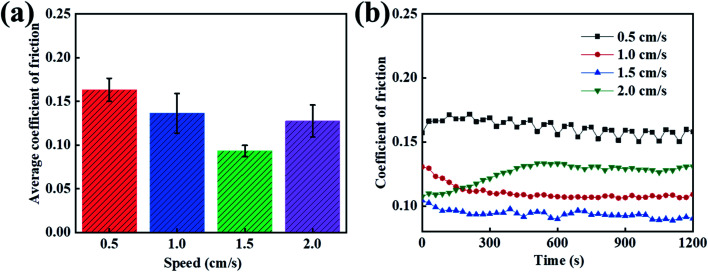
Curves of the different speeds of PVA/PEG/CFC. (a) Average coefficient of friction. (b) Coefficient of friction.

After studying the tribological properties of the different loads, the tribological properties of the carbon fiber composites are different at different velocities. In order to study the friction and wear properties of the PVA/PEG/CFC composites at different speeds, tribological tests were carried out at low and high speeds. Fig. 6(a) shows the average friction coefficient graphs at different speeds. According to the figure, the average coefficient of friction of the PVA/PEG/CFC composites decreases first and then it increases slightly with the increase in the sliding speed. When the speed is 1.5 cm s^−1^, the average coefficient of friction reaches a minimum of 0.8, which is approximately half of that at a low speed (1.5 cm s^−1^). In addition, the average coefficient of friction of the PVA/PEG/CFC composites decreases because the PEG on the surface of the CFC releases the lubricating material at a certain speed. As the speed continues to increase, the average coefficient of friction increases when the speed reaches 2.0 cm s^−1^ since the lubrication film on the surface of the CFC was destroyed due to the increase in the friction velocity at a high speed; hence, the average coefficient of friction increased. Fig. 6(b) shows the friction coefficient diagram of the PVA/PEG/CFC composites. It can be observed from the figure that with an increase in the friction time, the coefficient of friction at different speeds tends to be slightly stable, and as the speed continues to increase, the heat generated in the friction process can undergo convection with the air because of the carbon fiber's high thermal conductivity. This also reduces the coefficient of friction in comparison to the low-speed condition, and it reduces the heat accumulation or the fiber damage. Therefore, the composite material is not suitable for low- and high-speed conditions.

### 3.6 Wear surface analysis of PVA/PEG/CFC at different speeds

**Fig. 7:**
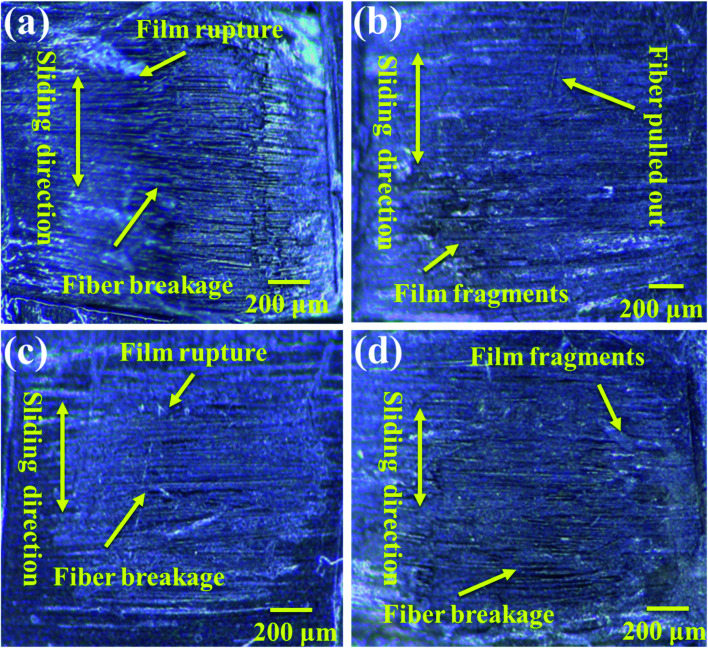
Wear surface analysis of PVA/PEG/CFC at different speeds: (a) 0.5 cm s^−1^, (b) 1.0 cm s^−1^, (c) 1.5 cm s^−1^, and (d) 2.0 cm s^−1^.

The friction and wear conditions at different speeds were investigated in order to further study the surface wear mechanism of the PVA/PEG/CF composites. The worn surface morphology at different speeds was observed and the friction time was 1 h. Fig. 7(a) shows the wear diagram at a friction speed of 0.5 cm s^−1^, and it can be observed that along the sliding direction, fiber fracture and lubrication film rupture are clearly observed on the surface. This is accompanied by a large number of fiber fragments, which indicates that the surface of the carbon fiber is seriously worn at low speed, and this is consistent with the highest average friction coefficient at a low speed. Fig. 7(b) shows the wear diagram for a friction velocity of 1.0 cm s^−1^, in which a part of the lubrication film is damaged on the surface of the PVA/PEG/CFC composite material, and a part of the friction fiber is fractured. Fig. 7(c) shows the wear diagram when the friction speed is 1.5 cm s^−1^ along the sliding direction. Along with the generation of scratches, the wear surface of the composite material is relatively neat, and it is accompanied by a small amount of fiber being pulled out. This is consistent with the lowest average coefficient of friction *t* at 1.5 cm s^−1^, which was previously proposed. Fig. 7(d) shows the wear diagram when the friction velocity is 2.0 cm s^−1^. Compared to the surface wear that is shown in Fig. 7(a), Fig. 7(d) shows that with an increase in the friction velocity, a large area of the lubrication film along with fracture of the carbon fiber can be observed. As a result, the lubrication film of the composite material was destroyed during friction, and the carbon fiber was exposed and rubbed directly with the friction ball. This led to an increase in the surface wear of the PVA/PEG/CFC composite material.

### 3.7 Analysis of the friction properties for different media

**Fig. 8:**
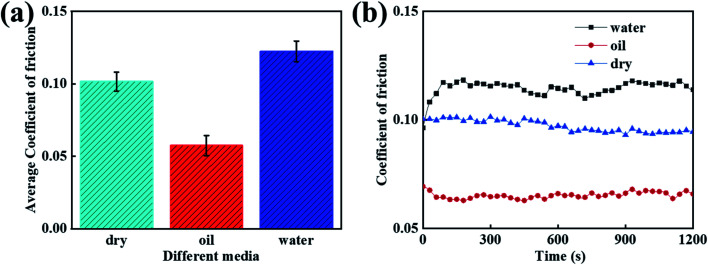
Curves of the different media of PVA/PEG/CFC. (a) Average coefficient of friction. (b) Coefficient of friction.

When considering different environments, the tribological properties of the composites are different. As a result, this investigation was focused on studying the tribological properties of the PVA/PEG/CFC composites under different lubrication environments, and the speed was set as 1.5 cm s^−1^. Fig. 8(a) shows the average coefficient of friction of the PVA/PEG/CFC composites under water, oil lubrication, and drying conditions. It can be demonstrated from the figure that oil, as a lubricant, plays a good role in lubrication during the friction process. Oil has an average coefficient of friction that is 0.05, which is approximately half of that under water lubrication conditions. The reason for this is that in a liquid environment, fluid lubrication can avoid direct contact between friction pairs, and the viscosity of oil is higher than that of water. In addition, oil as a lubricant can effectively reduce the average coefficient of friction of the composite materials. Therefore, in comparison to the dry and water lubrication conditions, the average coefficient of friction under oil lubrication conditions is the lowest. Fig. 8(b) shows the friction coefficient diagram of the PVA/PEG/CFC composites that are in different lubricating media. According to the figure, in comparison to the water lubrication condition, the friction coefficient under oil lubrication is reduced by approximately half; hence, the friction coefficient of the PVA/PEG/CFC composites is greatly reduced. With an increase in the friction time, the total friction coefficient is slightly stable. Therefore, the experimental results show that PVA/PEG/CFC is more suitable for oil lubrication.

### 3.8 Wear surface analysis of PVA/PEG/CFC at different media

**Fig. 9:**
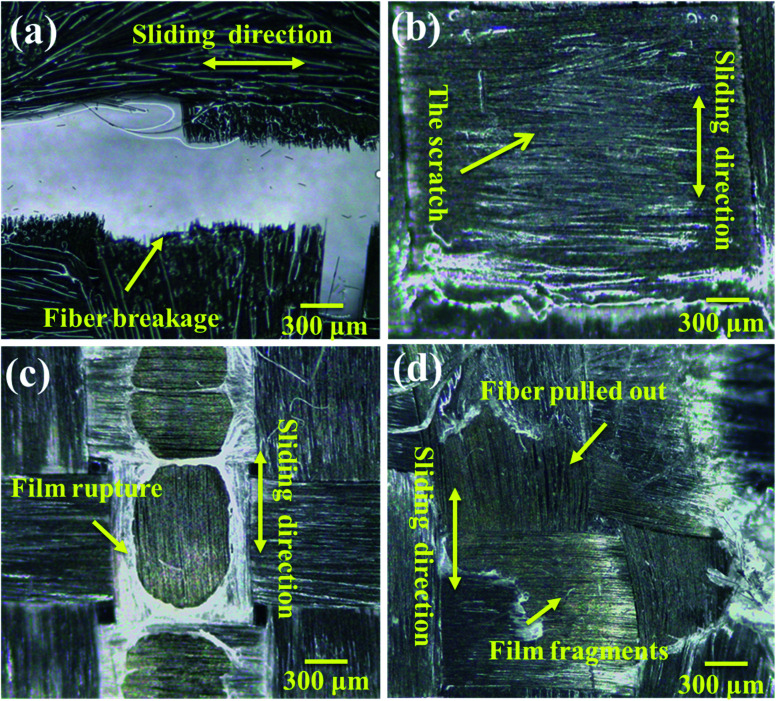
Surfaces of the PVA/PEG/CFC subjected to different lubricants, such as (a) raw carbon fiber/oil, (b) oil, (c) water, and (d) dry.

Based on the friction coefficient under the different lubrication conditions that are described above, in order to explore the wear condition of the PVA/PEG/CFC composite surface, the original carbon fiber fabric was used as the contrast experimental group, and the wear surface of the composite material under the different lubrication conditions was characterized by electron microscopy. Fig. 9(a) shows the surface wear diagram of the original carbon fiber fabric under oil lubrication. It can be observed that along the sliding direction, the entire carbon fiber has been broken and the fracture interface is neat. Fig. 9(b) shows the surface wear diagram of the PVA/PEG/CFC composite material under oil lubrication conditions, and it can be observed that the surface of the composite material has only slight friction scratches in the figure. Thus, it also proves the above prediction that the friction coefficient is the smallest in the oil lubrication environment and the surface wear of the composite material can be improved. Fig. 9(c) is the surface wear diagram of the PVA/PEG/CFC composite material under water lubrication. It is demonstrated that the surface of the composite material presents a gel-like protective film, and in the process of water friction, the film breaks and the fracture surface is relatively neat. In addition, a small amount of carbon fiber is pulled out. This is like a protective film since the residual PVA and PEG cross-linking can generate a soft gel; thus, resulting in a certain toughness in the lubrication film that is generated on the surface of the CFC. This kind of gel-like lubrication film is easy to soften and decompose when it encounters water; hence, the surface wear in the water lubrication environment is not good. Fig. 9(d) shows the surface wear diagram of the PVA/PEG/CFC composite material under dry friction conditions. It can be observed that the lubrication film on the fiber surface is broken and slightly coated on the fiber surface. This is accompanied by a small amount of fiber being pulled out. In conclusion, the surface wear diagram verified that the surface wear of the PVA/PEG/CFC composites was better when oil was used as a lubricant under different environmental conditions.

### 3.9 Friction analysis test of long time for PVA/PEG/CFC under oil lubrication

**Fig. 10:**
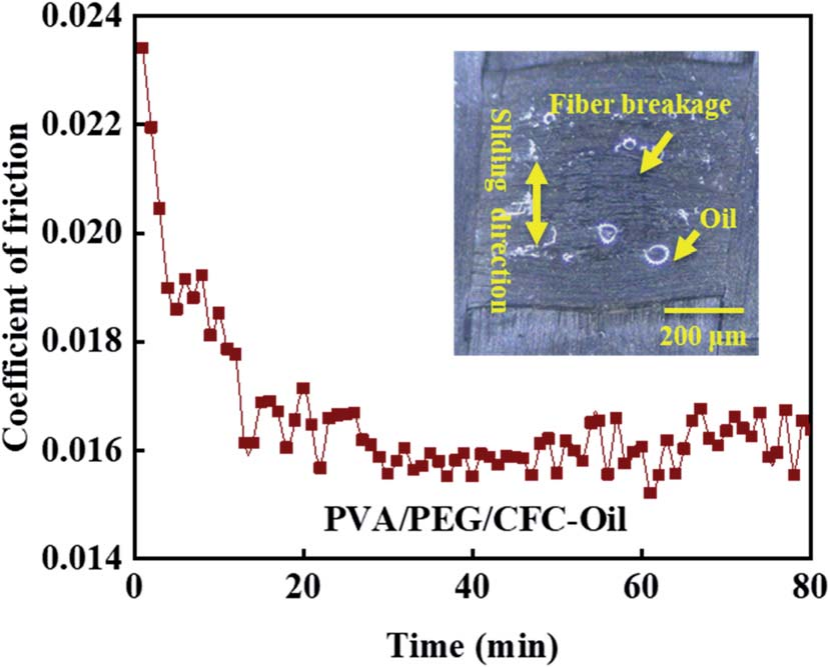
Long time friction test of PVA/PEG/CFC.

Considering the applications of carbon fiber composites in harsh environment, the experimental test shows that the composite material in the lubrication condition of friction performance is good, so combined with the practical application of CFC, a long time of oil lubrication friction test is necessary, as shown in Fig. 10, which shows the test for coefficient of friction of PVA/PEG/CFC composites under oil lubrication for up to 80 minutes. In the upper right corner of the figure, the surface wear light mirror image of the composite material is shown. The figure shows that coefficient of friction presents a gradually decreasing trend, which finally almost leveled off. During the friction process, the release of PEG in the lubrication film on the surface of PVA/PEG/CFC composites decreases the coefficient of friction. As the friction time continues to increase, the temperature of the composites increases, which accelerates the destruction of the lubrication film. Once the lubrication film is destroyed, the carbon fiber is inevitably exposed between the friction pairs. Due to the unique graphite structure of carbon fiber, carbon fiber has certain lubrication resistance to friction, so in the late friction period, the composite material presents a low coefficient of friction within a certain fluctuation range. At the same time, the surface wear image in the upper right corner shows that the carbon fiber breaks along the sliding direction of friction; although the surface wear of the composite material is poor in the process of long-term friction, its coefficient of friction still shows a low trend. Therefore, when operating under oil stain and bad environmental conditions, PVA/PEG/CFC composite material has a low friction coefficient, which can play an effective role in protecting the working parts.

### 3.10 Tensile fracture analysis of the different concentrations of PEG in PVA/PEG/CFC

**Fig. 11:**
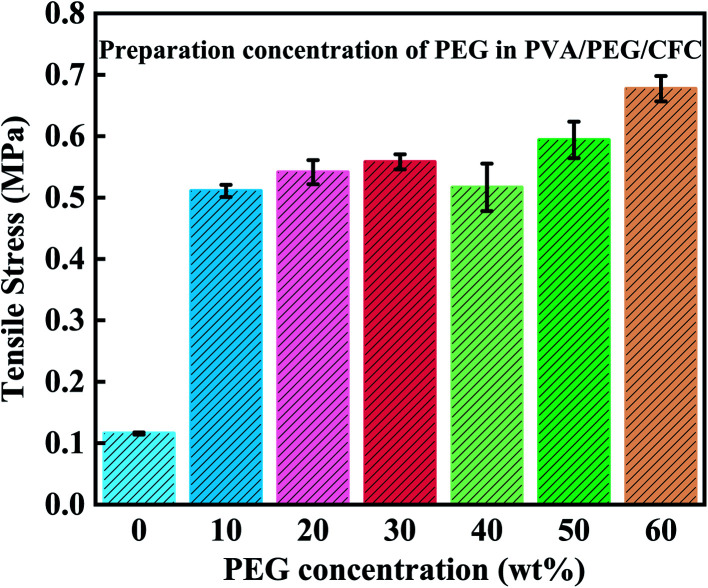
Tensile strength of PVA/PEG/CFC.

After studying the friction and wear, most of the mechanical properties of the composite material were understood. From this, the tensile strength of the composites was tested, as shown in Fig. 11. While preparing the PVA/PEG/CFC composites, different PEG concentrations had different tensile results in the experimental process, in which the original CFC was used as the control group. The tensile strength refers to the maximum stress of the material before tensile fracture, that is, the material's maximum resistance to plastic deformation. The figure shows that after treatment of the PVA/PEG composite materials, the tensile strength in comparison to the untreated CFC significantly enhances the mechanical strength.^31^ With an increase in the PEG concentration, the tensile strength of the composite materials shows a slightly smooth trend. The addition of PVA/PEG shows that the CFC forms a protective film layer. The protective film has a high toughness and it can withstand and transfer most of the applied load, it avoids direct exposure of the carbon fiber to the friction surface, and the surface mechanical properties of the composites are significantly improved. At the same time, due to the lubricity of PEG, the protective film presents a certain lubrication effect and slides in the stretching process, which results in a decrease in the tensile strength. However, with the increase in the PEG concentration, the increase in viscosity reduces the skid phenomenon and further increases the tensile strength.^40^

### 3.11 Analysis of the lubrication mechanism of PVA/PEG/CFC

**Fig. 12:**
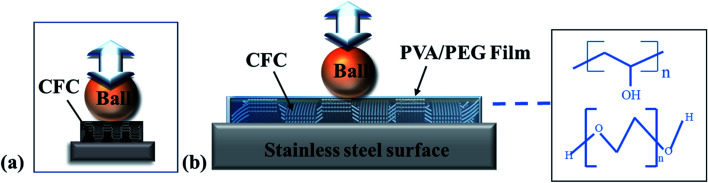
Wear mechanism of (a) raw carbon fiber and (b) PVA/PEG/CFC.

Based on the above friction test analysis and wear morphology observation, the wear mechanism of the PVA/PEG/CFC composites is proposed, and the results are shown in Fig. 12. It can be observed that the PVA/PEG/CFC composites are placed on the stainless steel surface. Afterwards, the steel ball was placed in the friction rod, which was then placed on the surface of the PVA/PEG/CFC composite material for the friction experiment, and it finally rotated at a constant speed. Fig. 12(a) shows the original wear diagram of the carbon fiber. It can be observed from the figure that the steel ball is in direct contact with the carbon fiber. By having an increase in the friction time, the stress concentration in the friction process leads to serious wear of the carbon fiber. Fig. 12(b) shows the wear pattern of the PVA/PEG/CFC composites. Because of the addition of PVA, a protective film with certain toughness was formed on the surface of the original CFC. It was easy to form a film on the PVA. As a result, the CFC has better mechanical strength, and the addition of PEG makes the protective film have a better lubricity, which is called lubricating film for short. Lubricating the film on the friction surface in the process of the composite materials under a certain load and speed will release a large amount of PEG, which lowers the average friction coefficient. However, with the increase in the load and speed, the rise in the speed of lubrication is significantly lower than the speed of friction, which results in rupture of the lubricating membrane. With the increase in the friction time, the carbon fiber is inevitably exposed in the friction pair; the carbon fiber and steel ball directly contact each other. Because of the graphite structure of the carbon fiber itself, it has some lubrication. From this, the coefficient of friction decreases at the beginning, but as time progresses, especially at a high speed and during heavy loading, it produces a lot of heat during friction and the lubrication film is broken, which results in a higher coefficient of friction.

## 4 Conclusions

In this study, the PVA/PEG/CFC composite materials were prepared by chemical crosslinking, and PVA/PEG was coated and disseminated on the surface of CFC to prepare a high-strength CFC with wear resistance. In addition, the mechanical and tribological properties under different concentration ratios, loads, and velocities were studied. The mechanism was analyzed through friction and wear tests under certain speed loading conditions, and the existence of PVA forms a protective film on the surface of the CFC, which improves the toughness of the CFC. In the friction process, when a certain external pressure load is applied, PEG will be released to play a certain lubrication role, direct contact between the carbon fiber and the steel ball can be reduced, and the friction coefficient is reduced. With the addition of PEG, the lubrication performance of the composite surface improved and the surface wear is reduced. Finally, the tensile test proved that the addition of PVA/PEG improved the mechanical properties and the tensile strength of the composites. At the same time, considering the hydrophilicity of PVA/PEG, it can be seen from the wear surface of the electron microscope that the damage of the lubrication film on the CFC surface is accelerated under the condition of water lubrication, which increases the surface friction and wear of the composite material. Meanwhile, the experimental results show that PVA/PEG has a good biocompatibility, and the material easily degrades under the water environment, thus making it a green and pollution-free composite. Although further research is needed to reduce the wear of the composite, the mechanical properties of the composites have also been significantly improved. However, long-term friction will aggravate the surface wear. XRD analysis showed that PVA/PEG/CFC composites had higher crystallinity, and the crystallinity affected the mechanical properties. TGA analysis showed that the presence of PVA/PEG could reduce the thermal decomposition rate, thus further improving the thermal stability of the composites.

## Author contributions

Ziqing Feng and Hailing Lu developed the initial concept. All authors discussed the results and commented on the manuscript.

## Conflicts of interest

The authors declare that they have no known competing financial interests or personal relationships that could have appeared to influence the work reported in this paper.

## Supplementary Material
